# Exploring the prognostic significance of lactate-mitochondria-related genes in prostate cancer

**DOI:** 10.3389/fgene.2024.1515045

**Published:** 2025-01-06

**Authors:** Yuan Wang, Ronghui Chen, Feng-Le Jiang, Xin Jiang, Yuehong Zhou, Yingying Zhou, Xinyi Hong, Chaoying Lin, Wei-Jia Wang, Sufang Qiu

**Affiliations:** ^1^ The school of Medicine, University of Electronic Science and Technology of China, Chengdu, China; ^2^ Clinical Oncology School of Fujian Medical University, Fujian Cancer Hospital (Fujian Branch of Fudan University Shanghai Cancer Center), Fuzhou, China; ^3^ Innovation Center for Cancer Research, Clinical Oncology School of Fujian Medical University, Fujian Cancer Hospital, Fuzhou, China; ^4^ Fujian Key Laboratory of Advanced Technology for Cancer Screening and Early Diagnosis, Fuzhou, China; ^5^ Fujian Key Laboratory of Translational Cancer Medicine, Clinical Oncology School of Fujian Medical University, Fujian Cancer Hospital, Fuzhou, China; ^6^ State Key Laboratory of Cellular Stress Biology, School of Life Sciences, Xiamen University, Xiamen, China

**Keywords:** prostate cancer (PCa), lactate-mitochondria-related genes (LMRGs), prognosis, myeloperoxidase (MPO), metastasis, drug sensitivity

## Abstract

Prostate cancer (PCa) is a common and serious health issue among older men globally. Metabolic reprogramming, particularly involving lactate and mitochondria, plays a key role in PCa progression, but studies linking these factors to prognosis are limited. To identify novel prognostic markers of PCa based on lactate-mitochondria-related genes (LMRGs), RNA sequencing data and clinical information of PCa from The Cancer Genome Atlas (TCGA) and the cBioPortal database were used to construct a lactate-mitochondria-related risk signature. Here, we established a novel nine-LMRG risk signature for PCa, and Kaplan-Meier curves confirmed a worse prognosis for high-risk subgroups in the TCGA dataset. Meanwhile, a nomogram that effectively predicts the prognosis of PCa patients was also constructed. Next, close associations between the lactate-mitochondria-related signature and the immune microenvironment were examined to clarify the role of LMRGs in shaping the immune landscape. Furthermore, as the only lactate-related gene among the nine key prognostic risk genes, myeloperoxidase (MPO) was identified as a key factor that mediates lactate production *in vitro* and *in vivo* through attenuation of the glycolytic pathway. More importantly, MPO significantly inhibited PCa cell migration, invasion, and epithelial–mesenchymal transition (EMT), indicating its potential as an anticancer gene. Additionally, PCa with high MPO expression is highly sensitive to chemotherapeutic agents and mitochondrial inhibitors, highlighting its potential as an improved therapeutic strategy for PCa management.

## Introduction

Prostate cancer (PCa) is a malignant tumor that originates from the epithelial cells of the prostate gland, and cause significant mortality among elderly men. Currently, approximately 1.4 million new cases and 0.4 million deaths are reported annually (Bray et al., 2024). Although advancements in technology have significantly enhanced the early diagnosis of PCa, the survival rate has not significantly improved, because of the high rate of bone metastasis and insensitive to treatment. Radical prostatectomy (RP) or radiotherapy is the first-antineoplastic treatment for patients in early stage with localized PCa (Siegel et al., 2017). However, 30%–50% of patients will still progress to biochemical relapse after taking treatment (Lalonde et al., 2014). Around 20% of intermediate-risk patients face biochemical failure within 18 months of initial local treatment (Shao et al., 2009; Nichol et al., 2005). The oncogenic mechanisms that drive PCa are not yet well understood, making it challenging to implement targeted therapy for high-risk PCa and castration-resistant prostate cancer (CRPC) (Xie et al., 2018; Li et al., 2020). Therefore, gaining a deeper understanding of the various characteristics of PCa and identifying of effective prognostic indicators are essential for developing more effective treatment strategies for PCa.

Metabolic reprogramming is a acknowledged hallmark of cancer and involves pathways such as glycolysis, oxidative phosphorylation, and mitochondrial metabolism (Li et al., 2020; Mohsen et al., 2019; Delaunay et al., 2022). This shift in metabolic processes of cancer cells enables them to satisfy their heightened energy requirements, support rapid growth, and survive under various stress conditions. Additionally, cancer cells often modulate the levels of metabolic byproducts, such as lactate, to influence the tumor microenvironment, thereby affecting the behavior of both cancer cells and immune cells (Sarah et al., 2020). This intricate interplay between metabolic pathways and cellular interactions is critical for tumor development and progression, and thus this interplay is a focal point for potential therapeutic interventions.

Traditionally, lactate has been considered a metabolic waste product excreted by glycolytic prostate cancer cells into the microenvironment (Pereira‐Nunes et al., 2020). However, with deeper research, it has been discovered that lactate serves not only as the preferred energy substrate for PCa cells but also plays a role in reprogramming their metabolism through interactions with cancer-associated fibroblasts (CAFs) (Pierre et al., 2008; Fiaschi et al., 2012). Metabolic reprogramming in PCa is frequently associated with altered mitochondrial function, as mitochondria are not only the site of oxidative phosphorylation but are also the central hub of multiple metabolic pathways, including the tricarboxylic acid cycle and fatty acid oxidation (Mamouni et al., 2021). Many studies suggest that, metabolic reprogramming is associated with changes in mitochondrial bioenergetics, biogenesis and dynamics during PCa developmentt (Vikramdeo et al., 2023; Haokun et al., 2024; Xiao et al., 2018). The increase in ROS and sulfide oxidation flux, along with the reduction of ATP generation, may exacerbate PCa malfunction and lead to higher grade malignancy (Bee et al., 2021). Elucidation the intricate molecular mechanisms of mitochondrial-participated metabolic reprogramming and exploration the role of these mechanisms in carcinogenesis progression are essential for identifying innovative therapeutic targets and strategies for PCa.

Lactate metabolism, which promotes tumor cell proliferation and metastasis via modulating the tumor microenvironment, enhancing angiogenesis, and suppressing the immune response, is also particularly critical for cancer progression. Lactate is a byproduct of glycolysis and is especially enriched in rapidly growing tumors. Accumulated studies have confirmed that lactate serves as a high-energy substrate that shuttles between the cytoplasm (glycolysis) and mitochondria (oxidative phosphorylation) (Daniel and Kane, 2014; Safer et al., 1971; George et al., 1999). Lactate participates in mitochondrial oxidative reactions through the lactate-malate-aspartate shuttle and functions as an energy substrate that enhances energy support and regulates androgen metabolism; therefore, lactate potentially offers new therapeutic avenues for PCa (Daniel and Kane, 2014; Glancy et al., 2021).

Although numerous studies have demonstrated the importance of lactate and mitochondrial function on tumor progression, the specific genes related to the association between these two pathways that could serve as prognostic markers for PCa remain unclear. This knowledge gap hinders the development of precise prognostic tools and effective therapeutic strategies. This study aimed to explore and identify LMRGs with prognostic significance in PCa. We conducted a comprehensive bioinformatics analysis using data from large-scale PCa studies. Differentially expressed genes were identified via statistical methods, and survival ratio analysis was conducted to assess their prognostic significance. We established a novel nine-LMRG signature and accompanying nomogram that can accurately forecast the outcome of PCa patients. Furthermore, we demonstrated that myeloperoxidase (MPO), a lactate metabolism-related gene, plays a critical role in mediating lactate production by modulating the glycolytic signaling pathway, which results in significant inhibition of EMT, migration, and invasiveness of PCa cells. Additionally, PCa cells with higher levels of MPO expression show more sensitivity to chemotherapeutic agents and mitochondrial inhibitors, which highlights its potential to improve the current therapeutic strategies for PCa management.

## Materials and methods

### Data collection

Fragments per kilobase transcript (FPKM) data and corresponding clinical information of PCa patients were obtained from the TCGA-PRAD dataset of TCGA database (https://portal.gdc.cancer.gov), which includes 52 normal samples and 502 tumor samples. Clinical data (Supplementary Table S1) were obtained from cBioPortal’s collection of clinical data from PCa patients (https://www.cbioportal.org). External clinical data of TCGA-PRAD (Supplementary Table S2) were obtained from the UCSC Xena platform (https://xenabrowser.net/datapages/). The data were processed and analyzed via Perl software (version Strawberry-perl-5.30.0.1; https://www.perl.org) and the R Bioconductor package in R software (version R-4.4.1).

### Screening of lactate and mitochondria-related genes

The list of lactate-related genes (Supplementary Table S3) was compiled from relevant literature on lactate-associated gene sets (Jiang et al., 2023). The list of mitochondria-associated genes presented in Supplementary Table S4 was compiled from well-curated datasets (Chang et al., 2023), including the MitoCarta 3.0 database (Rath et al., 2020) and the molecular signatures database (MSigDB) (Mootha et al., 2003; Subramanian et al., 2005). Differentially expressed genes (DEGs) between PCa and normal tissues were identified using the “limma” package in R. Genes were considered significantly differentially expressed in PCa samples relative to normal tissues if they had an absolute log2-fold change (logFC) greater than 0.585 (equivalent to a fold change exceeding 1.5) and a false discovery rate (FDR) below 0.05.

### Development of prognostic risk features based on LMRGs

To further refine the DEGs, we applied a univariate Cox regression analysis with a p-value <0.05 as the selection criterion to identify the genes as lactate-mitochondria-related markers. To avoid overfitting, we employed the “glmnet” and “survival” packages for LASSO Cox regression analysis (Friedman et al., 2010). Following LASSO regression, multivariate Cox regression analysis was performed to establish LMRGs based on the selected markers. The entire cohort was randomly divided into training and testing groups at a 1:1 ratio for internal validation. Patients were classified into high-risk and low-risk categories on the basis of the risk scores from the training, testing, and overall groups via median split values. The risk score was calculated according to the following formula:

$$\text{Risk Score}=\sum_{i=1}^{n}\left(\text{Ge}\text{neexp}\times \text{Coef}i\right)$$

Here, “n” represents the number of mRNAs associated with PCa prognosis, and “i” denotes the *i*th LMRG. The expression levels of LMRGs and the regression coefficients are represented by Geneexp and Coefi, respectively. The risk score for each patient was predicted using the “predict” function included in the “survival” R package. Patients were divided into two subgroups, the LMRG-high-risk subgroup and the LMRG-low-risk subgroup, according to the median LMRG score.

### Validation of prognostic risk features

The prognostic value of the LMRGs was assessed via Kaplan‒Meier (KM) survival analysis, which compared the progression-free survival (PFS) rates between the two LMRG groups from the TCGA database. We further explored the model’s predictive capabilities concerning clinical variables such as age, T stage, N stage, and the risk score. To ensure the robustness of the model, multivariate independent prognostic analysis was conducted, and ROC curves were generated for these clinical features using the “timeROC,” “survival,” and “survminer” R packages. In addition, we used prognostic data from TCGA-PRAD, including disease-free interval (DFI), disease-specific survival (DSS), and overall survival (OS), for external validation to evaluate the model’s generalizability.

### Immune microenvironment evaluation

Following the global immune classification of solid tumors developed by (Thórsson et al., 2018), we identified the following four distinct immune subtypes: C1 (wound healing), C2 (IFN-γ-dominant), C3 (inflammatory), C4 (lymphocyte-depleted). To determine the relationship between risk scores and immune phenotypes, the “ggpubr” R package was utilized. To evaluate immune cell infiltration in PCa patients, both the “CIBERSORT” and “xCell” R packages were utilized. “CIBERSORT” estimated the abundances of 22 specific immune cell types, while “xCell” provided a broader analysis, assessing 64 distinct cell types based on gene expression data. The immune-related characteristics were defined according to the previous studies, and the scores were calculated via gene set variation analysis (GSVA).

### Drug sensitivity calculation

We used the “OncoPredict” (Maeser et al., 2021) R package to evaluate the potential clinical applications of LMRGs in PCa treatment. OncoPredict predicts drug response according to the RNA-Seq gene expression data. Specifically, we utilized the calcPhenotype function to estimate the drug response curves (IC50 values) for commonly used chemotherapeutic drugs via baseline tumor gene expression data from the TCGA database. The Wilcoxon signed-rank test was then employed to compare the IC50 values between different LMRG risk groups to identify statistically significant differences.

### Functional enrichment

To examine the activity of key pathways and identify differences between the high- and low-risk groups, gene set variation analysis (GSVA) was conducted. The R package “clusterProfiler” (Wu T. et al., 2021) was employed to conduct Gene Ontology (GO) and Kyoto Encyclopedia of Genes and Genomes (KEGG) pathway enrichment analyses on the nine-LMRG, showcasing the top 10 results. These results were visualized using bubble charts. The GO analysis covered terms related to biological processes (BP), cellular components (CC), and molecular functions (MF).

### Antibodies and reagents

Anti-MPO (Cat# 11117-1-AP) antibody was purchased from Proteintech. Anti- FAK (Cat# 3285), anti-pFAK Tyr925 (Cat# 3284), anti-Snail (Cat# 3879), anti-E-Cadherin (Cat# 3195) and anti-actin (Cat# 4970) antibodies were purchased from Cell Signaling Technology. Goat anti-rabbit (Cat# 31210) and anti-mouse (Cat# 31160) secondary antibodies were purchased from Thermo Fisher Scientific. Docetaxel (Cat# HY-B0011) and paclitaxel (Cat# HY-B0015) were purchased from MedChemExpress. antimycin A (Cat# A8674) was purchased from Sigma-Aldrich.

### Cells and cell culture

Normal prostate cells WPMY-1, RWPE1, RWPE2, and prostate adenocarcinoma cells C4-2, LNCaP, PC3, and DU145 were obtained from Xiamen Immocell Biotechnology Co., Ltd. (Xiamen, China). These cells were cultured in Dulbecco’s Modified Eagle’s Medium (DMEM) (Sigma Aldrich, St. Louis, United States) or in RPMI-1640 medium (Sigma Aldrich, St. Louis, United States), according to the supplier’s specifications. Both media were supplemented with 10% fetal bovine serum (FBS) (Genial Biologicals, Inc., Brighton, United States). Regular *mycoplasma* tests confirmed no contamination.

### Western blot

Cells were lysed using ELB lysis buffer containing both protease and phosphatase inhibitor cocktails. After lysis, the samples underwent centrifugation at 14,000 × g for 15 min at 4°C. The supernatants obtained were mixed with 2 × SDS sample buffer and heated at 95°C–100°C for 5–10 min to ensure denaturation. Subsequently, the samples were applied to SDS-PAGE gels for electrophoresis, transferred onto PVDF membranes, and then analyzed via immunoblotting using specific antibodies.

### Generation of the lentiviral system

The oligonucleotides for short hairpin RNAs (shRNAs) were subcloned into the lentiviral vector pLL3.7, and expressed in LNCaP cells. First, lentiviruses were generated in HEK293T cells through co-transfection with the pLL3.7 vector carrying shRNA sequences, packaging plasmids, and polyethylenimine (PEI) for 48 h. After harvesting the viral supernatants, viruses were concentrated by centrifuging at 75,000 × g for 1.5 h, and subsequently filtered through 0.45 μm pore-size membranes (Millipore). The LNCaP cells were infected with the freshly isolated lentiviruses, and knockdown efficiency was tested using reverse transcription PCR (RT-PCR) after 48 h of incubation. The oligonucleotide sequences for the construction of the shRNA-targeted mRNAs were listed as below: Non-Targeting Control (NTC)-shRNA, 5′- CCT​AAG​GTT​AAG​TCG​CCC​TCG-3′; MPO-shRNA-1, 5′- GCC​ATG​GTC​CAG​ATC​ATC​ACT-3′; MPO-shRNA-2, 5′- GCA​GTA​CAC​TTC​CTG​CAT​TGA-3′; MPO-shRNA-3, 5′- GGT​TAT​GTG​TAT​GTG​CCA​TTT-3′.

### Extracellular acidification rate (ECAR) assay

To assess the extracellular acidification rate of the cells, a Glycolysis Stress Test Kit from Agilent Technologies (Santa Clara, CA, United States) and a Seahorse XFe96 Extracellular Flux Analyzer were employed. In brief, 10 (Shao et al., 2009) cells were plated per well in Seahorse XF96 cell microplate and incubated for 24 h. Glucose (10 mM), followed by the oxidative phosphorylation inhibitor oligomycin (1 μM) and the glycolysis inhibitor 2-DG (50 mM), were sequentially injected into each well. The Seahorse XFe96 instrument was used to capture the dynamic fluorescence signals. Finally, all the data were normalized to the cell count.

### Cell cycle detection

To detect the cell cycle, cells were initially collected and rinsed with PBS. They were then fixed in 70% ethanol, followed by another PBS wash. Afterward, the cells were stained with propidium iodide (PI) for 30 min in darkness. Flow cytometry was used to analyze the stained cells.

### *In vitro* cell migration and invasion assays

*In vitro* cell migration experiments were conducted using 8-mm polyester Transwell chambers (Corning, New York, United States). For cell invasion assays, a method akin to the migration assay was followed, as previously detailed by Wang et al. (Wang et al., 2019). The protocol was similar to that used for the migration experiments, except that the chambers were coated with growth factor-reduced Matrigel beforehand. Briefly, LNCaP cells were placed, in triplicate, into the Transwell chambers at a density of 2 × 10 (Nichol et al., 2005) cells per well in 0.1% BSA RPMI 1640 medium. As a chemoattractant, conditioned medium from NIH3T3 cells was collected and added to the lower chamber. Following a 16-h incubation period, any non-invading or non-migrating cells were removed from the upper membrane surface. The cells that had migrated or invaded to the underside of the Transwell insert were then fixed with methanol and stained with crystal violet. Images of five random fields at ×10 magnification were captured for each membrane and analyzed using ImageJ software.

### Metabolite analysis by LC‒MS

A total of 5 × 10^6 cells were thoroughly rinsed 3 times with cold PBS (4°C). Metabolites from each sample group were extracted using 1.6 mL of 80% methanol chilled to −80°C. The extracts, along with the cells, were placed into 2 mL tubes, subjected to vortex mixing for 1 min, and then centrifuged at 140,00× g and 4°C for 10 min. The supernatants were dried using a vacuum centrifuge (Labconco Corporation). The dried samples were reconstituted with 200 μL of 50% acetonitrile. A volume of 2 μL from each sample was injected into a QTRAP 5500 mass spectrometer (SCIEX) connected to a UPLC system (AB Sciex, ExionLC AD system), and separated on a ZIC-pHILIC column (SeQuant, 5 μm, 100 × 2.1 mm, Merck). For the mobile phases, buffer A was made up of 15 mM ammonium acetate with the pH adjusted to 9.7 using ammonium hydroxide, while buffer B contained 90% acetonitrile. The column was kept at 40°C and the samples at 10°C. A flow rate of 0.2 mL/min was maintained, with the gradient set as follows: 95% B from 0 to 2 min, 45% B from 15 to 18 min, and then back to 95% B from 18 to 22 min. The QTRAP instrument was operated in negative ion mode using multiple reaction monitoring (MRM).

### Lactate detection

Lactate release in the cell culture supernatants was analyzed using Lactate-Glo assay kits (Promega Corp., Madison, WI, United States) according to the manufacturer’s protocols. To quantify lactate production, the culture medium was diluted at a 1:20 ratio with PBS and then plated in 96-well plates. An equivalent amount of lactate detection reagent, which includes reductase, lactate dehydrogenase, reductase substrate, luciferin detection solution, and NAD, was added to the wells. The plates were incubated at room temperature for 1 h, after which luminescence was measured and normalized to the count of viable cells.

### MTT assay

Briefly, a total of 6 × 10³ LNCaP cells were plated in triplicate in a 96-well plate and treated with the indicated reagents in a final volume of 200 μL per well for a duration of 72 h at 37°C. Cells receiving DMSO acted as the control group. After then, 10 μL of MTT solution (5 mg/mL) was added to each well, and the plates were incubated for another 4 h at 37°C. Afterward, the MTT-containing medium was discarded, and 150 μL of DMSO was re-added to each well to dissolve the formazan crystals. The plates were incubated for an additional 10 min, and the absorbance at 490 nm was then recorded using a microplate reader.

### Statistical analysis

**FIGURE 1:**
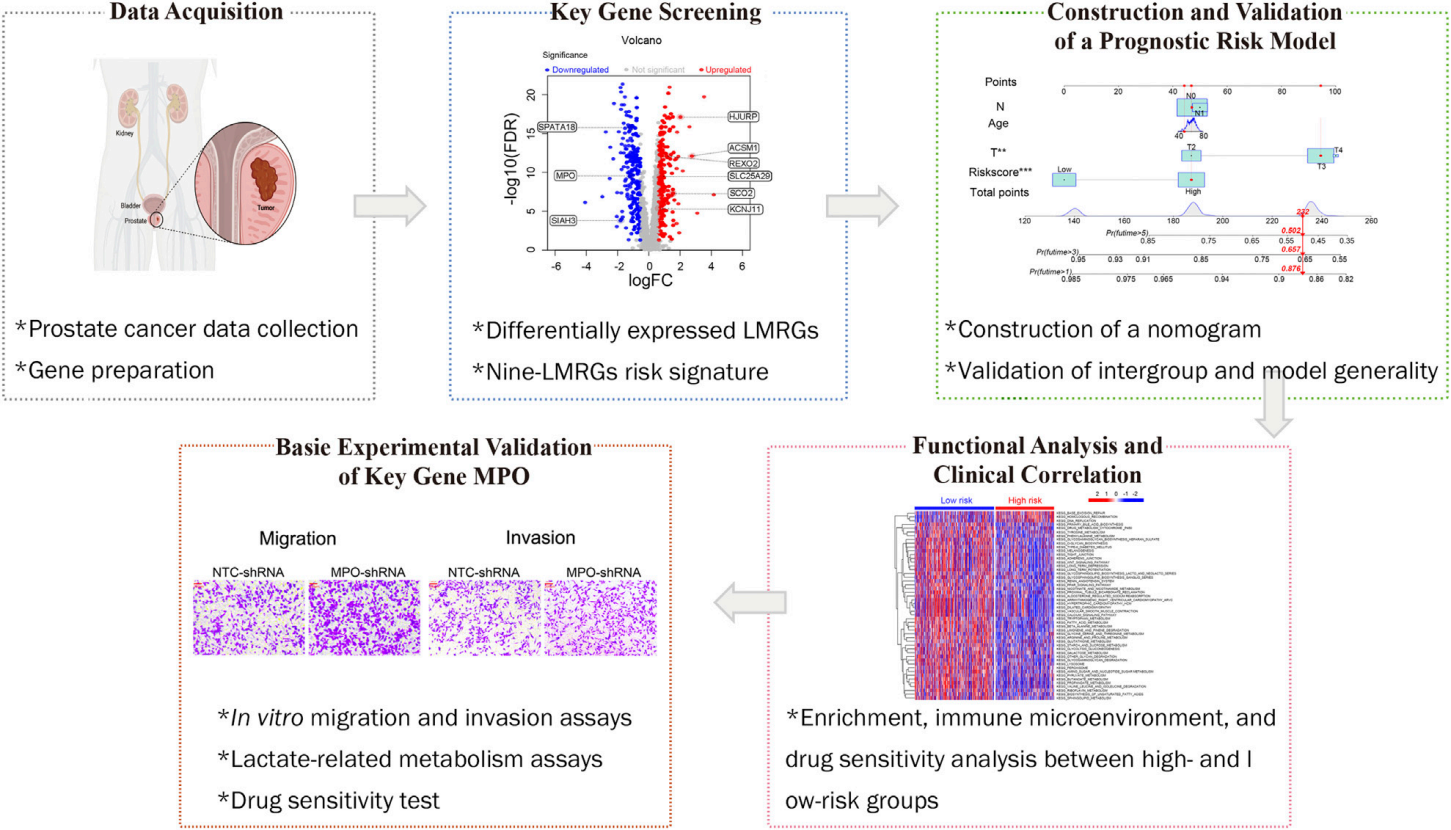
Research process flowchart.

Differential expression box plots for MPO were obtained from TNMplot (Bartha and Győrffy, 2020) (accessed on 15 August 2024). The differences in the proportions of clinical characteristics were analyzed via the chi-square test. Differences between KM curves were assessed via the log-rank test. A *p*-value less than 0.05 indicated statistical significance. Statistical analyses were performed via R or GraphPad software. The current study investigated the publicly available data, and no ethical approval was required. The logical flow of the study is illustrated in Figure 1.

## Results

### Identification of lactate-mitochondria-related differentially expressed genes

**FIGURE 2:**
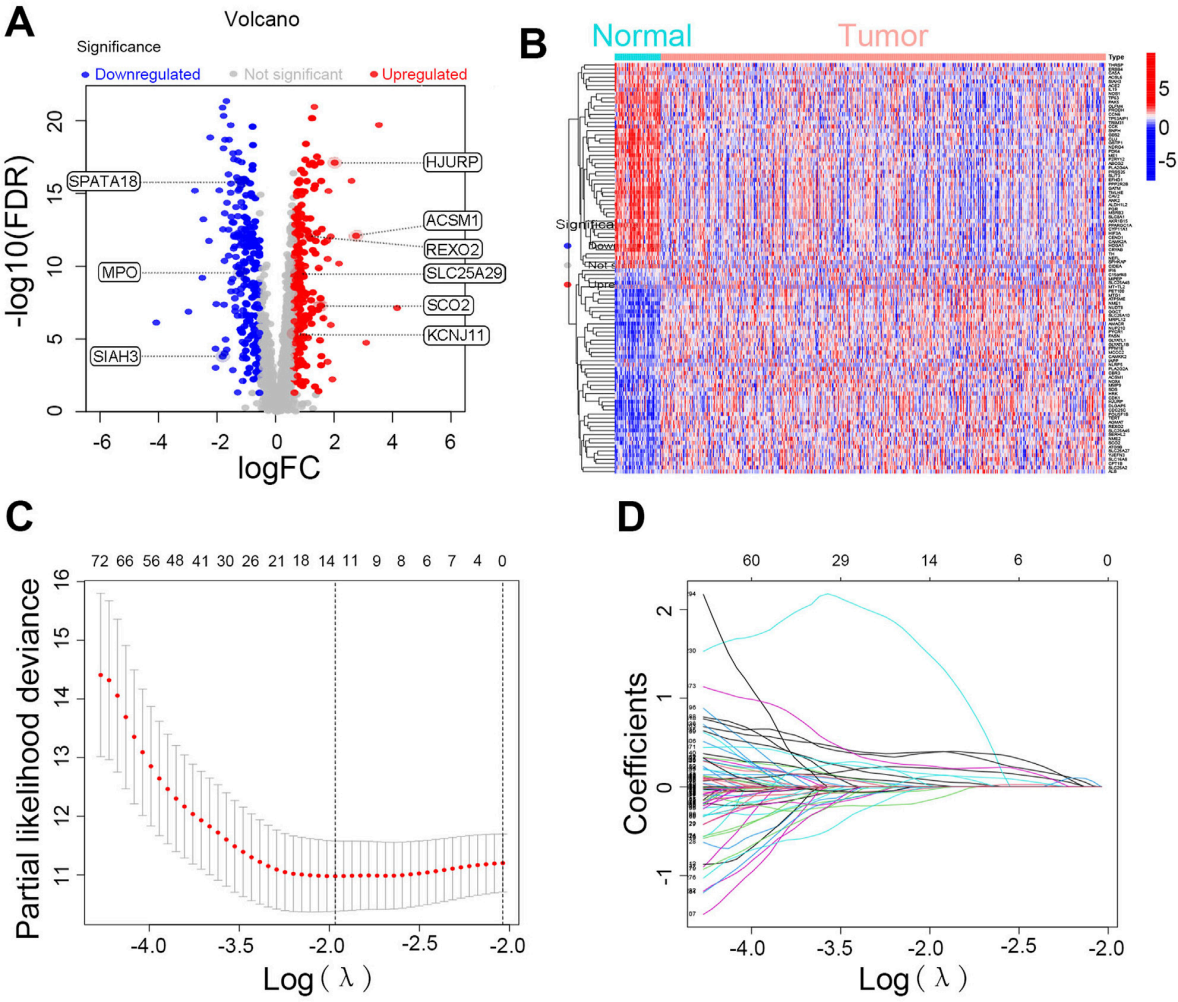
Identification of differentially expressed LMRGs and construction of prognostic risk model. **(A)** Volcano plot and **(B)** heatmaps of differentially expressed genes between PRAD and normal prostate tissue of LMRGs. **(C)** The LASSO regression coefficient spectrum. **(D)** Cross-validation of parameter selection in the LASSO model.

We initially integrated 206 lactate-related genes and 2,030 mitochondria-related genes to form a LMRG set. Figure 1 shows the workflow of the LMRG signature analysis. Briefly, RNA-seq data of 52 normal samples and 502 prostate adenocarcinoma (PRAD) samples were downloaded from the TCGA database. In addition, the clinical characterization and prognostic data of 494 TCGA-PRAD patients from cBioPortal were integrated, and we censored the data showing NA. As shown in the volcano plot (Figure 2A), 443 lactate-mitochondria-associated DEGs were identified in the TCGA-PRAD dataset (Supplementary Table S5). From these, the top 50 upregulated genes and the top 50 downregulated genes were selected according to the logFC and visualized in a heatmap (Figure 2B).

### Construction of prognostic genes and a risk model for LMRGs

We integrated patient survival data and performed univariate regression analysis, using a p-value below 0.05 as the statistical threshold to determine genes significantly linked to survival rates. Among 443 DEGs, 145 LMRGs showed significant associations with prognosis (Supplementary Table S6). Further analysis was performed on the 145 LMRGs using LASSO and multivariate Cox regression (Figures 2C, D), ultimately resulting in the construction of a prognostic risk signature based on nine-LMRGs. The risk score was calculated using the following formula:

$$\text{Risk Score}=\left[\text{MPO expression}\right]\times \left(-1.9763\right)+\left[\text{KCNJ}11\text{ expression}\right]\times \left(-0.5236\right)+\left[\text{SPATA}18\text{ expression}\right]\times \left(-0.3166\right)+\left[\text{ACSM}1\text{ expression}\right]\times \left(-0.2261\right)+\left[\text{HJURP expression}\right]\times \left(0.3196\right)+\left[\text{REXO}2\text{ expression}\right]\times \left(0.4176\right)+\left[\text{SLC}25A29\text{ expression}\right]\times \left(0.5170\right)+\left[\text{SCO}2\text{ expression}\right]\times \left(0.9966\right)+\left[\text{SIAH}3\text{ expression}\right]\times \left(2.5355\right)$$

### Validation of the risk model

**FIGURE 3:**
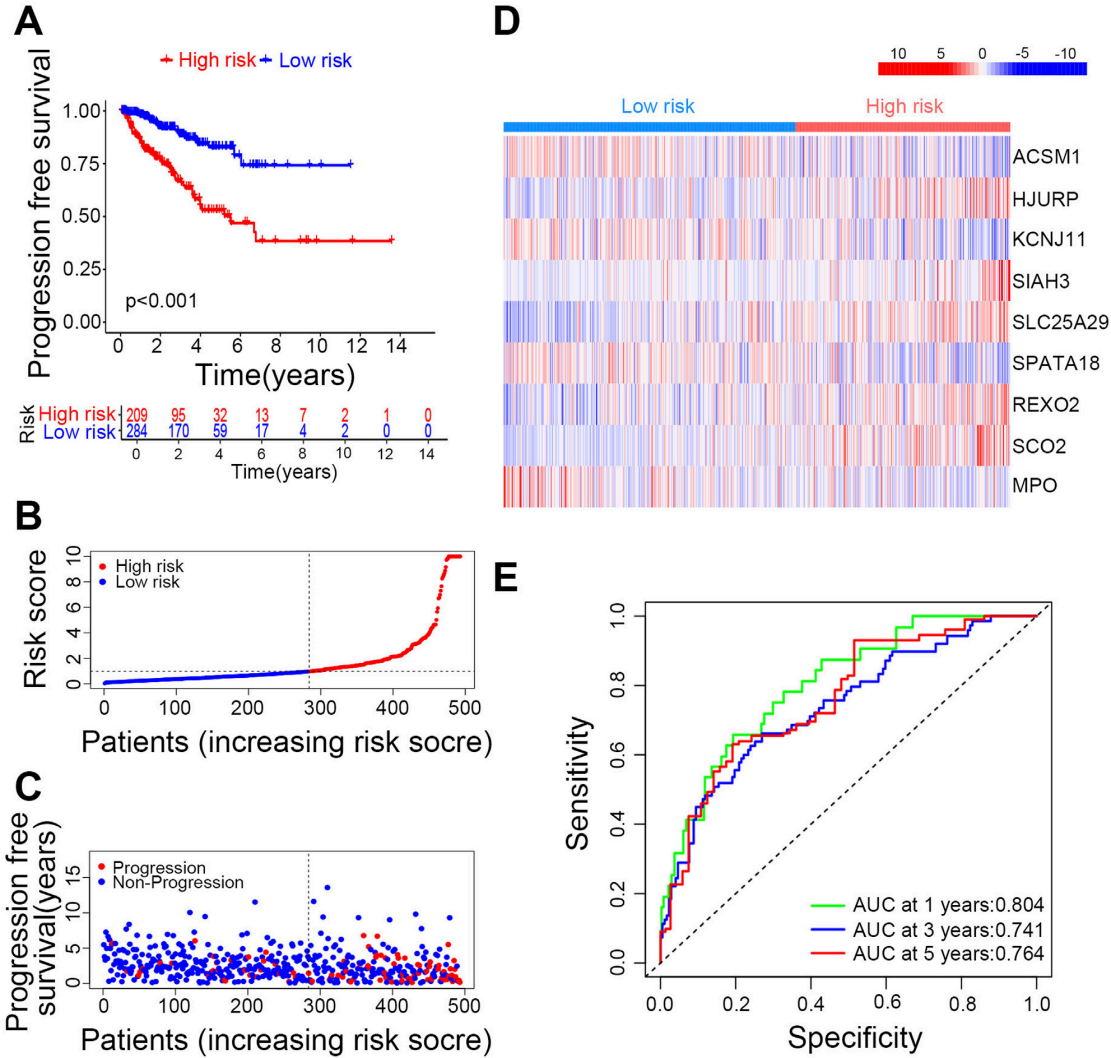
Validation of the prognostic risk model in the overall group. **(A)** Overall group recurrence survival curves. **(B)** Risk scores and group distribution in the overall group. **(C)** Recurrence status distribution map. **(D)** Frequency distribution of key genes in high- and low-risk groups. **(E)** ROC curves for 1, 3, and 5 years.

First, the aforementioned Risk Score model was used to score the PRAD samples. Based on the median risk score, the samples were classified into high-risk and low-risk groups. The entire cohort with accessible clinical data (n = 416) was then randomly split into a training set (n = 210) and a testing set (n = 206) in a 1:1 ratio, showing no significant differences in clinical characteristics between the groups (*P* > 0.05, Supplementary Table S7). Patient prognosis was effectively differentiated between the high-risk and low-risk groups, with the low-risk group consistently showing better outcomes (Figure 3A). For survival analysis, patients received risk scores and were categorized into high-risk and low-risk groups. Overall, higher risk scores correlated with increased recurrence rates in PCa patients (Figures 3B, C). In the low-risk group, genes ACSM1, KCNJ11, SPATA18, and MPO were highly expressed, while the other five key genes showed high expression in the high-risk group (Figure 3D). The area under the ROC curve (AUC) values for 1, 3, and 5-year predictions were all above 0.740 (Figure 3E). We validated similar outcomes in both the training and testing sets (Supplementary Figures S1A–J), confirming the model’s accuracy and effectiveness. To assess the model’s generalizability, we conducted an extensive analysis using prognostic data for disease-free interval (DFI), disease-specific survival (DSS), and overall survival (OS) from TCGA-PRAD via the UCSC Xena platform (Supplementary Figures S2A–C). The findings show that the risk model accurately predicts PCa recurrence and is applicable to various other prognostic scenarios, underscoring its potential as a reliable tool for evaluating survival outcomes in PCa patients.

### Independent prognostic value of risk features

**FIGURE 4:**
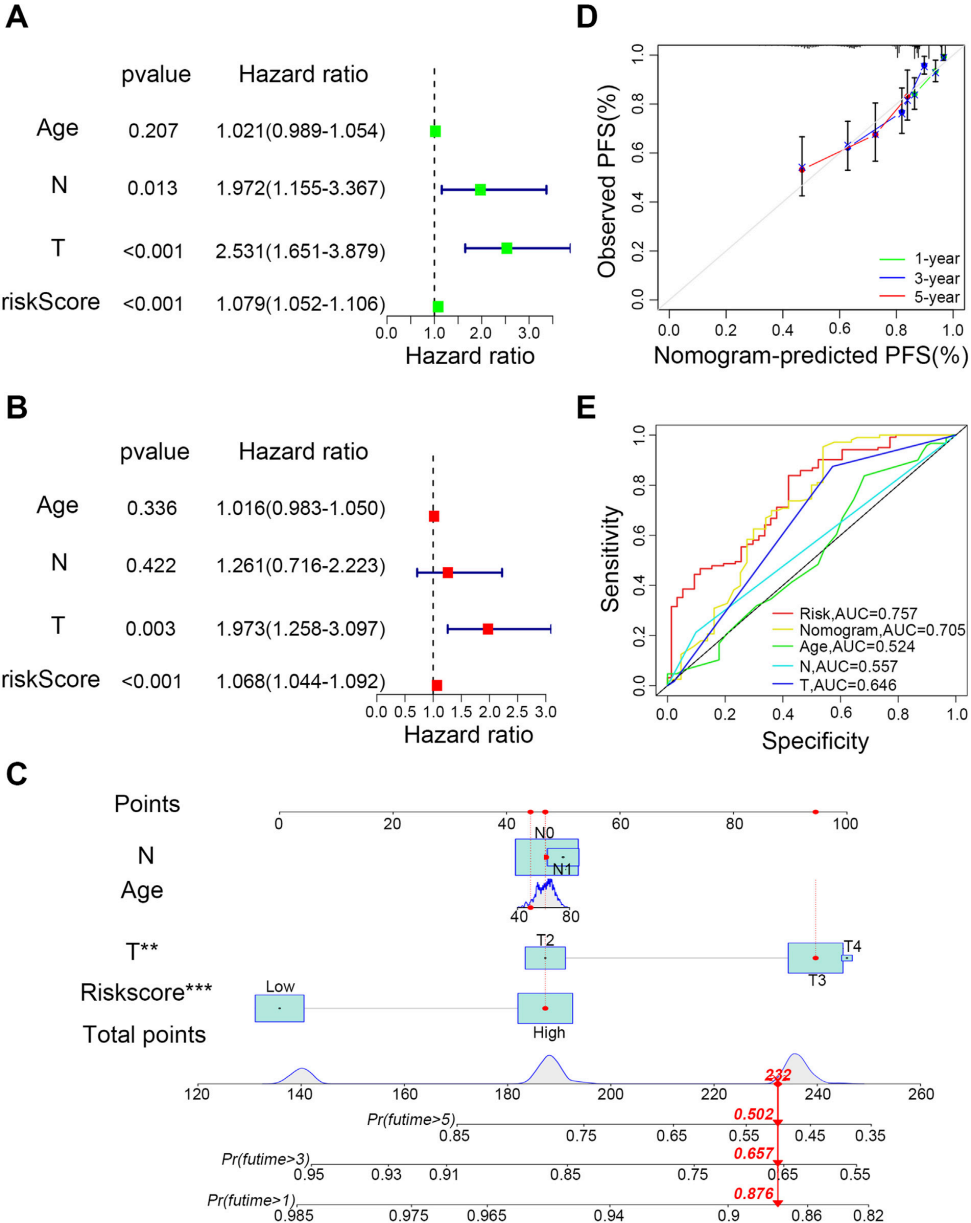
Determination of the effectiveness of the prognostic risk model. **(A)** Univariate and **(B)** multivariate forest plots of risk scores. **(C)** Construction of the nomogram. **(D)** Calibration curves for recurrence at 1, 3, and 5 years. **(E)** Comparison of the predictive effectiveness of the risk score and nomogram with other clinical characteristics; ***P < 0.001, **P < 0.01, *P < 0.05.

To assess the prognostic predictive power of the nine-LMRGs risk signature and other clinical factors for PCa, univariate (Figure 4A) and multivariate Cox regression analyses (Figure 4B) were performed. The results showed that the risk signature met the significance threshold (p < 0.05) in both analyses, which indicates that the nine-LMRGs risk signature has superior predictive ability compared with known clinical factors such as age, T stage, and N stage. According to the above analyses, we constructed a nomogram using the prognostic risk signature to predict the 1-, 3-, and 5-year survival probabilities of patients (Figure 4C). To assess the reliability of the model, 1-year, 3-year, and 5-year calibration curves were plotted. The result showed that the points closely aligned with the standard line, indicating a high degree of accuracy and consistency between the predicted and observed outcomes (Figure 4D). Additionally, AUC curves of the risk model and nomogram were compared with those of other clinical features, and the results showed that the nine-LMRGs risk signature was the strongest predictor than those of other clinical characteristics (Figure 4E). These results highlight the superior effectiveness of the risk model in predicting patient prognosis.

### Differences in immunity and drugs sensitivity between risk groups

**FIGURE 5:**
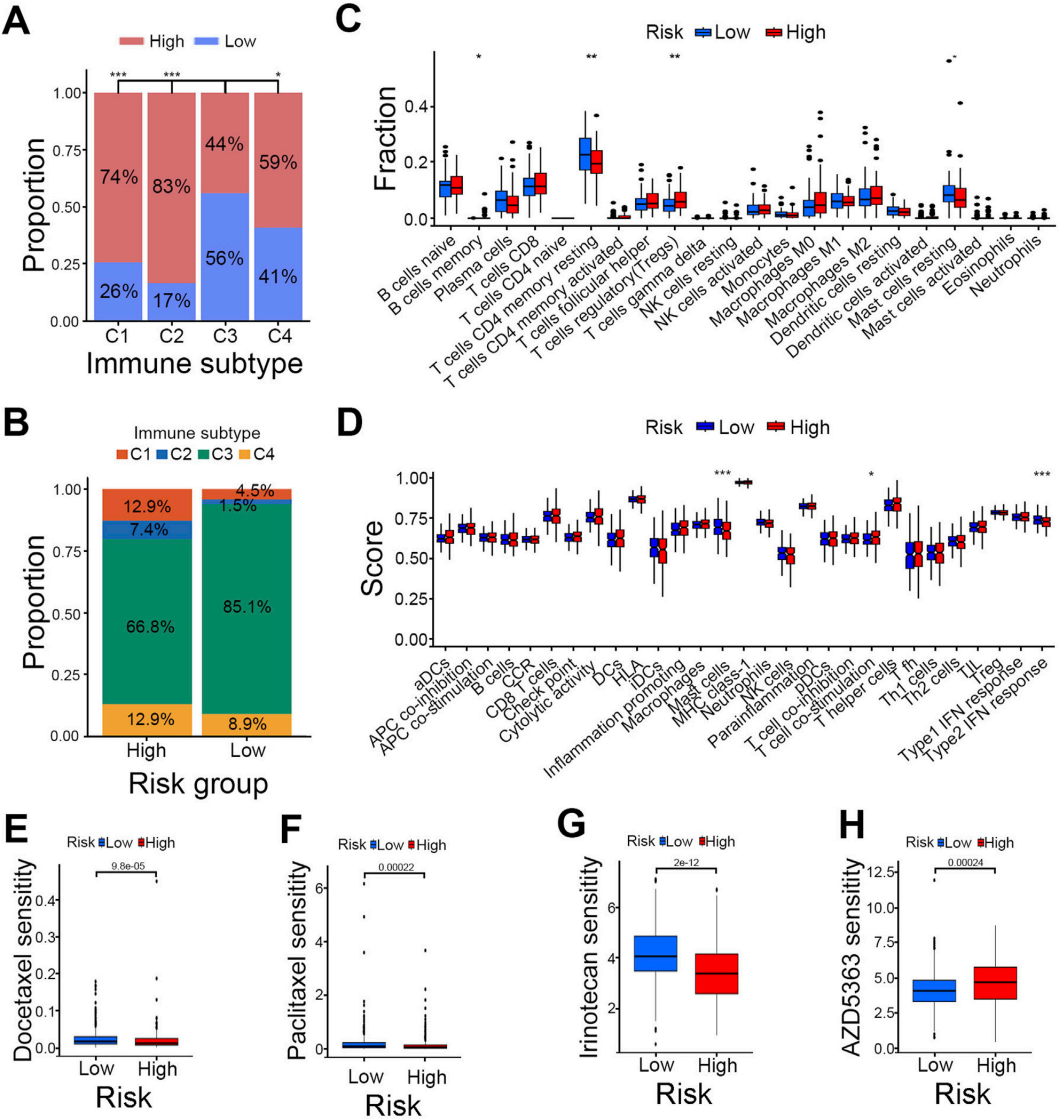
Comparison of immune profiles and drug sensitivity between risk groups. **(A)** Intergroup comparison of immune subtypes. **(B)** Proportions of immune subtypes in high- and low-risk groups. **(C)** Immune cell correlation of risk groups. **(D)** Immune function correlation of risk groups. **(E–H)** Drug sensitivity analysis of Docetaxel **(E)**, Paclitaxel **(F)**, Irinotecan **(G)** and AZD5363 **(H)**; ***P < 0.001, **P < 0.01, *P < 0.05.

Then we explored the correlation between risk scores and previously reported immune subtypes. Correlation analysis revealed that the C3 (inflammatory) subtype was significantly different from the C1 (wound healing), C2 (IFN-gamma-dominant) and C4 (lymphocyte-depleted) subtypes (Figure 5A) and had the highest immune infiltration rate in both high- and low-risk groups (Figure 5B). Moreover, the frequencies of the C1, C2, and C4 subtypes were higher in the high-risk group compared to the low-risk group (Figure 5B). To delve deeper into the associations between PCa risk groups and immune cell infiltration, we utilized the CIBERSORT algorithm. This analysis demonstrated that the infiltration of regulatory T cells (Tregs) was significantly higher in the high-risk group, whereas the infiltration levels of memory B cells, resting memory CD4 T cells, and resting mast cells were markedly lower compared to the low-risk group (Figure 5C). To pinpoint specific immune function subtypes that are activated in PCa, we conducted further analysis of the correlation between immune functions and risk scores using the CIBERSORT algorithm. The results showed that in the low-risk group, immune functions were significantly linked to mast cells, while T-cell co-stimulation was more pronounced in the high-risk group (Figure 5D).

We then assessed the sensitivity of high- and low-risk groups to common anticancer drugs to find potential treatment strategies for PCa. The findings indicated that the low-risk group responded better to clinical chemotherapy and targeted therapies, such as docetaxel, paclitaxel, and irinotecan (Figures 5E–G). Meanwhile, the high-risk group showed greater sensitivity to the AKT inhibitor AZD5363 (Figure 5H). These insights offer a theoretical foundation for selecting clinical drugs in PCa treatment.

### Enrichment analysis of key LMRGs and pathway comparison between groups

To further investigate the genes comprising the risk model, we conducted GO functional enrichment analysis and KEGG pathway enrichment analysis on the nine-LMRGs. As shown in the results, the BP terms were involved in non-membrane-bounded organelle assembly, muscle system process, and muscle tissue development. The CCs were mainly enriched in sarcomere, myofibril, and contractile fiber, whereas the MFs were enriched predominantly in actin binding, hormone activity, and structural constituent of muscle (Supplementary Figure S3A). The KEGG enrichment results highlighted pathways related to the cytoskeleton in muscle cells and motor proteins (Supplementary Figure S3B).

We subsequently performed GSVA to explore potential pathways differentiating the risk groups. The results indicated that the high-risk group was enriched primarily in the BASE EXCISION REPAIR, HOMOLOGOUS RECOMBINATION, and DNA REPLICATION pathways, whereas the low-risk group was enriched in the GLYCOLYSIS GLUCONEOGENESIS, PROPANOATE METABOLISM, and PYRUVATE METABOLISM pathways (Supplementary Figure S3C). The enrichment of these pathways in the high-risk group suggests a focus on responding to DNA damage and maintaining genomic stability, possibly due to oxidative stress and mitochondrial dysfunction caused by lactate accumulation, which exacerbates DNA damage. In contrast, the low-risk group was enriched in pathways related to glycolysis/gluconeogenesis, propanoate metabolism, and pyruvate metabolism, emphasizing the maintenance of mitochondrial energy metabolism and metabolic homeostasis, thus preventing excessive lactate accumulation. Based on pathway analysis, the results revealed that lactate serves as a key factor in associating mitochondrial-related functions and influences genomic stability as well as metabolic pathways between the high- and low-risk groups. Therefore, we selected the lactate-related genes for further investigation.

### MPO inhibits lactate production in PCa

**FIGURE 6:**
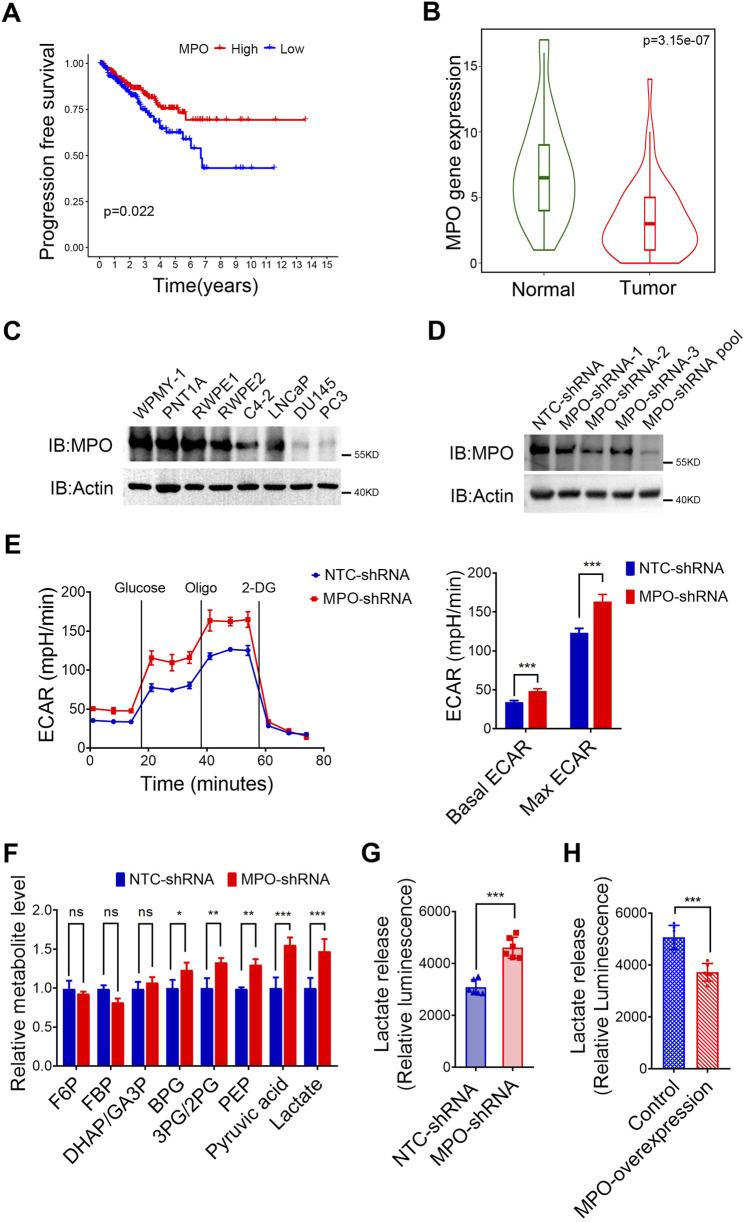
MPO inhibits lactate production in PCa. **(A)** Univariate survival analysis of MPO in TCGA-PRAD. **(B)** Differential gene expression analysis of MPO in the TNM-Ploter. **(C)** MPO protein levels in PCa cell lines. **(D)** The endogenous MPO expression in LNCaP cells was knocked down using a lentiviral system. Actin was used to determine the amount of loading proteins. **(E)** The ECAR of LNCaP cells was detected. **(F)** Intracellular metabolites were extracted from LNCaP cells, and glycolytic metabolites were measured by LC-MS. **(G, H)** The level of extracellular lactate in the medium of LNCaP cells **(G)** and PC3 **(H)** was detected. All data are presented as the mean ± SD of three independent experiments, ***P < 0.001, **P < 0.01, *P < 0.05.

As the only lactate-related gene among the 9 key prognostic risk genes, MPO was identified as a protective factor according to the univariate KM survival curve (Figure 6A). Additional analysis via the TNMplot revealed that MPO expression in PCa tissues was significantly lower than that in normal tissues (Figure 6B). We further discovered that MPO expression levels are significantly associated with the infiltration of various immune cells, including monocytes and dendritic cells (Supplementary Figures S4A, B). We further examined MPO expression levels in PCa cell lines with varying degrees of malignancy. Compared with normal prostate cells (WPMY-1, PNT1A, RWPE1, RWPE2) and poorly metastatic PCa cells (C4-2, LNCaP), moderately metastatic PCa cells (DU145, PC3) presented lower levels of MPO expression (Figure 6C).

MPO selectively oxidizes thiol-containing proteins, particularly those involved in the glycolysis pathway, which leads to the disruption of basal glycolysis (Love et al., 2016). To further confirm this, we established stable MPO knockdown LNCaP PCa cells, after which the knockdown efficiency was measured by Western blot analysis (Figure 6D). As demonstrated in Figure 6E, MPO silencing resulted in increases in both the basal and maximum ECARs in LNCaP cells. Moreover, as shown in Figure 6F, the levels of glycolytic metabolites, particularly pyruvate and lactate, were elevated in the absence of MPO, which supports its role in the inhibition of glycolysis. In addition to increasing the ECAR, the absence of MPO also led to increased extracellular lactate release (Figure 6G). We further overexpressed MPO in PC3 cells, where endogenous MPO is barely detectable. Our study demonstrated that MPO overexpression significantly reduced extracellular lactate release, as expected (Figure 6H). In conclusion, our data confirm that MPO is not only linked to the lactate pathway but that it also significantly suppresses glycolysis and lactate production.

### MPO inhibits prostate cancer metastasis

**FIGURE 7:**
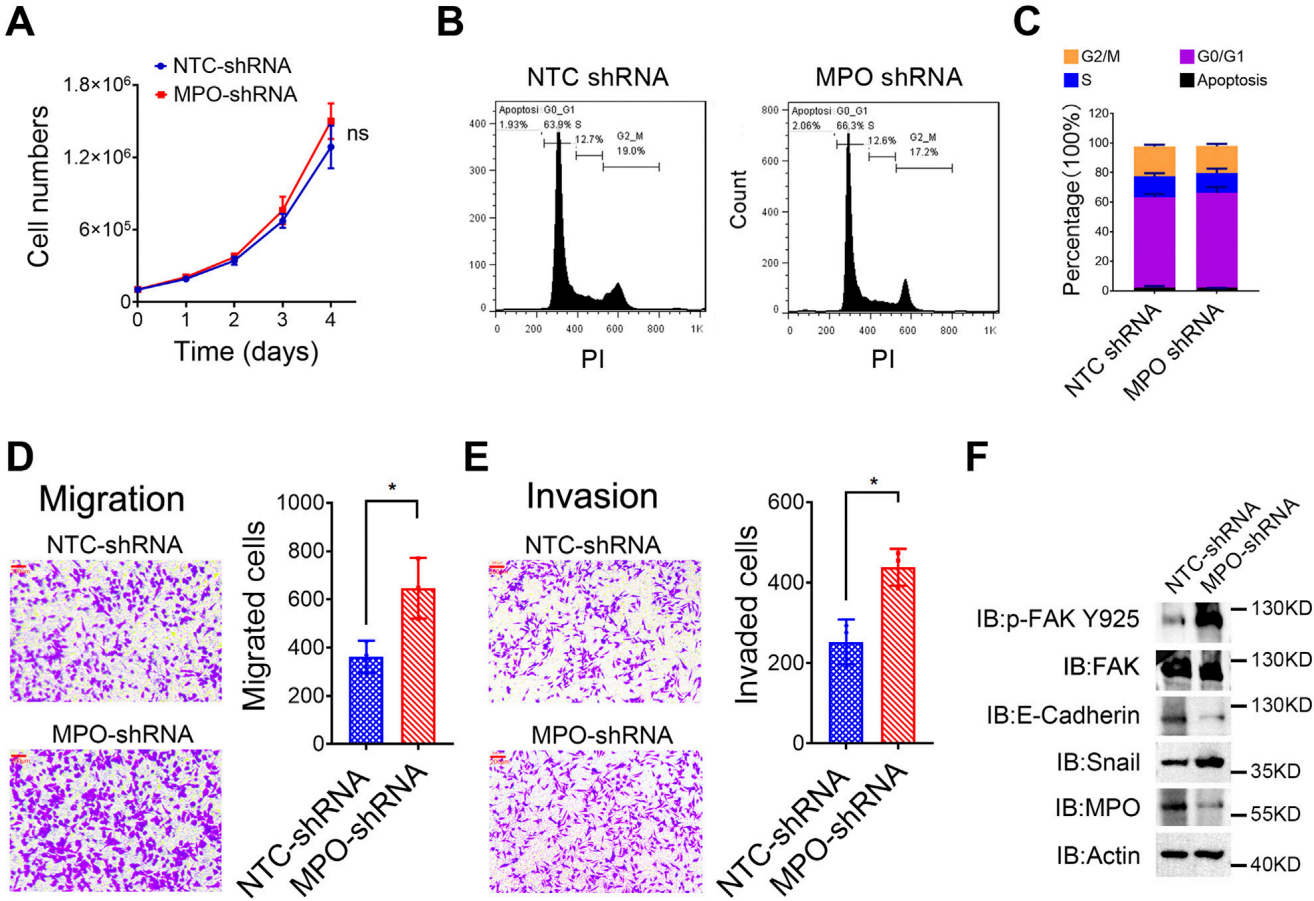
MPO inhibits prostate cancer metastasis. **(A)** The cell number of LNCaP cells was counted at different time points. **(B, C)** Cell cycle analysis through PI staining was detected by FACS **(B)**, and the cell cycle distribution was presented **(C)**. **(D, E)** LNCaP cells were analyzed for cell migration and invasion. Representative images of crystal violet-stained migrated **(D)** or invaded **(E)** cells are presented (scale bar, 100 μL). **(F)** The Tyr925-phosphorylated level of FAK and total FAK, E-Cadherin, and Snail in LNCaP cells were detected by Western blot analysis. Actin was used as a loading control. All data are presented as the mean ± SD of three independent experiments; ns, not significant.

We subsequently explored the role of MPO in PCa. Our results indicated that silencing MPO did not significantly impact cell proliferation (Figure 7A) or cell cycle progression (Figures 7B, C). However, MPO knockdown via shRNA markedly enhanced the migration (Figure 7D) and invasion (Figure 7E) abilities of LNCaP cells. These results imply that MPO plays more pivotal role in regulating metastasis than in influencing cell proliferation in PCa.

Focal adhesion kinase (FAK) is a tyrosine kinase situated at extracellular matrix adhesion sites and is crucial for cell motility. Furthermore, E-cadherin and Snail are well-known biomarkers of EMT. LNCaP cells with MPO-shRNA exhibited increased total protein levels of Snail and higher phosphorylation of FAK at the Tyr925 site, along with a reduced protein level of E-cadherin (Figure 7F). Collectively, these findings demonstrate that MPO is crucial for metastasis modulation in PCa LNCaP cells.

### PCa with high MPO expression is highly sensitive to drugs

**FIGURE 8:**
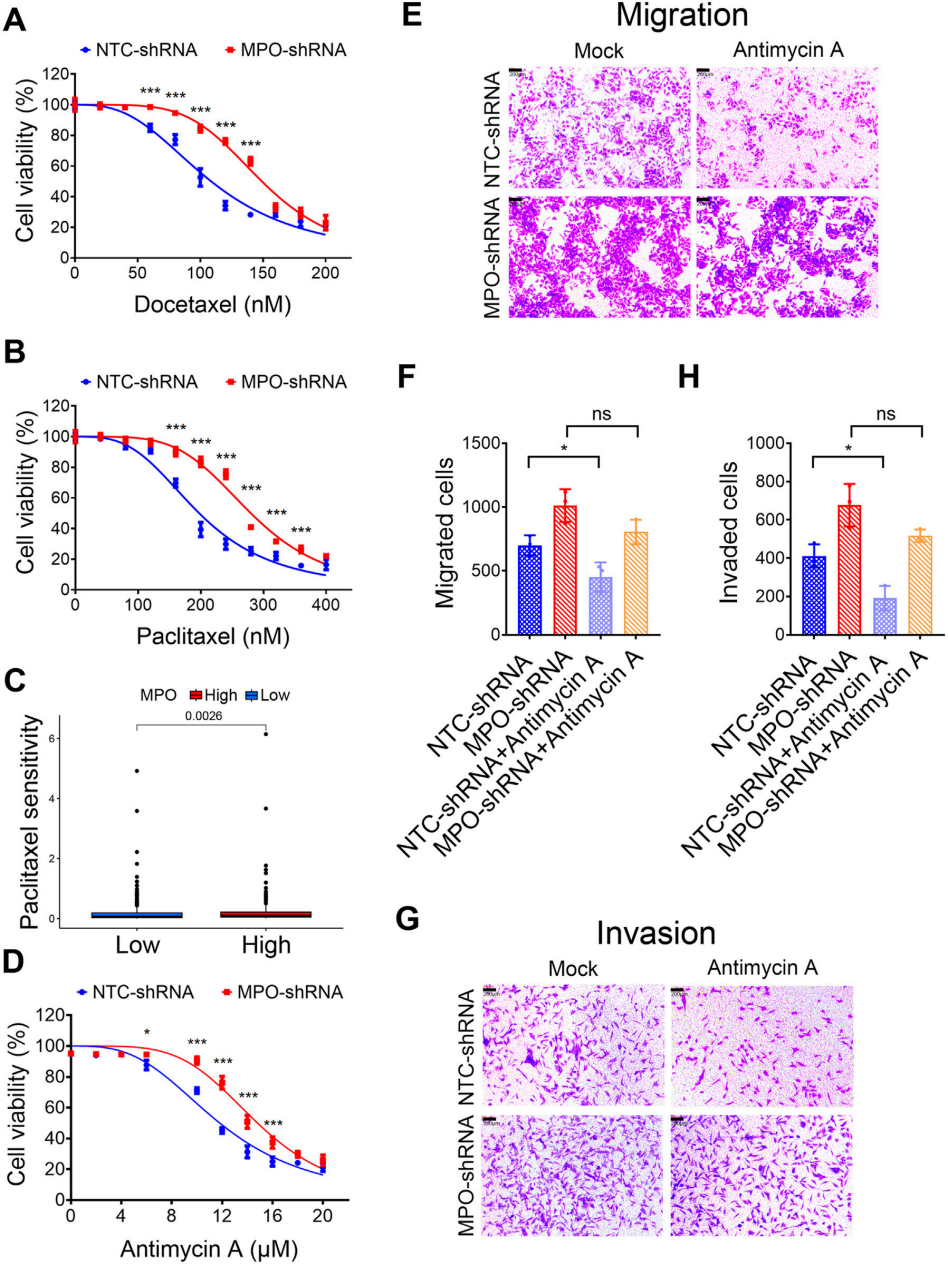
PCa with high MPO expression exhibits higher drug sensitivity. **(A, B)** LNCaP cells were treated with docetaxel **(A)** or paclitaxel **(B)** for 72 h, and then cell viability was determined by MTT assays. **(C)** Drug sensitivity analysis of TCGA-PRAD to paclitaxel. **(D)** LNCaP cells were treated with Antimycin A for 72 h, and then cell viability was determined by MTT assays. **(E–H)** LNCaP cells were treated with the Antimycin A (10 μM) and analyzed for cell migration and invasion. Representative images of crystal violet-stained migrated **(E)** or invaded **(G)** cells are presented (scale bar, 100 μL). Quantification for migrated cells **(F)** or invaded cells **(H)** are presented. All data are presented as the mean ± SD of three independent experiments; ***P < 0.001, *P < 0.05; ns, not significant.

To investigate whether MPO affects PCa cell sensitivity to clinical chemotherapy, we knocked down the expression of MPO in LNCaP cells. Comparing with the cells in control group, the cells in MPO silencing group lose the sensitivity to docetaxel (Figure 8A) and paclitaxel (Figure 8B). Moreover, overexpression of MPO significantly increased sensitivity to docetaxel (Supplementary Figure S5A) and paclitaxel (Supplementary Figure S5B). According to drug sensitivity analysis in TCGA-PRAD, it was found that high MPO expression is associated with increased sensitivity to paclitaxel (Figure 8C). This is consistent with our experimental conclusions.

We then investigated, whether different expression levels of MPO influence the reliance of PCa cells on mitochondrial metabolism/oxidative phosphorylation. Antimycin A is wildly used for mitochondrial dysfunction, which blocks mitochondrial complex III (cytochrome bc1 complex) of the respiratory chain via disrupting electron transport and ATP synthesis (Tzung et al., 2001). LNCaP control cells are vulnerable to mitochondrial inhibitors. Compared with them, LNCaP cells with silenced MPO exhibited decreased sensitivity to antimycin A (Figure 8D). Similarly, antimycin A significantly inhibited both migration and invasion, whereas MPO knockdown diminished this inhibitory effect on migration and invasion (Figures 8E–H). These findings suggest a close relationship between mitochondrial function and the glycolytic pathway, which is characterized by lactate production. Therefore, targeting both pathways could offer an effective approach for the treatment of PCa.

## Discussion

PCa is the most common malignant cancer among men in Western countries, with an incidence rate that continues to increase (Bray et al., 2024; Sung et al., 2021). Despite the approval of abiraterone and enzalutamide, mortality has only slightly decreased (Bono et al., 2011; Howard et al., 2012). The deaths of PCa patients can be broadly categorized into cancer causes and noncancer causes. The risk of death due to noncancer causes is influenced by factors such as stage, ethnicity, and treatment variations (Guo et al., 2022; Guo et al., 2020). The incidence and mortality rates of PCa among African American men in the United States are significantly higher than those among White men, by at least 1.7 times (Siegel et al., 2022). While genetic predisposition contributes to the increased incidence of PCa in African Americans, social factors play a more critical role in prognosis. Studies have shown that African American and white men with similar PCa stages can achieve comparable outcomes when provided with equal access to healthcare (Rana et al., 2020; Bergengren et al., 2023; Dess et al., 2019) Uncontrollable factors such as age, family history, and germline variations also pose significant threats to survival (Bergengren et al., 2023).

Aberrant metabolism in PCa, which involves the regulation of various metabolic pathways, has been identified as a key factor in disease progression and metastasis (Chen et al., 2023). Lactate is recognized as a crucial signaling molecule in cellular metabolism. Lactate produced by cancer cells is secreted into the extracellular environment, where it induces tumor progression by affecting the tumor microenvironment (Certo et al., 2020; Ippolito et al., 2019). In addition to being a byproduct of energy metabolism, lactate can act as a signaling molecule that regulates mitochondrial dynamics (Zhou et al., 2024). As organelles involved in biosynthesis and energy generation, mitochondria enable cells to rapidly adapt to their environment and are considered important mediators of tumorigenesis. Recent experimental studies have confirmed the importance of balancing mitochondrial function and glycolysis in cancer metastasis (Delaunay et al., 2022). Therefore, a comprehensive investigation into the roles of lactate and mitochondria-related genes in the pathogenesis and prognosis of PCa is urgently needed.

In this study, we identified and validated LMRGs that play a significant role in the prognosis of PCa patients. By integrating large-scale RNA-seq data from the TCGA-PRAD dataset, we screened and analyzed DEGs associated with lactate metabolism and mitochondrial function. Among the 443 identified DEGs, we focused on 145 genes significantly related to prognosis. Ultimately, we developed a prognostic risk model based on LMRGs via LASSO and multivariate Cox regression analysis. Our prognostic risk model effectively stratified patients into high-risk and low-risk groups, with significant differences in PFS observed between the two groups. The model demonstrated its validity not only in PFS but also in OS, DSS and DFI outcomes. To explore the underlying mechanisms, we analyzed the differences in immune and pathway activities between risk groups. The GSVA results revealed strong associations between different risk groups and various metabolic pathways involving lactate and mitochondria, which further confirms their critical role in PCa prognosis. Additionally, GO and KEGG enrichment analyses of risk-related genes highlighted key cellular functions and pathways involved. The analysis of immune cell infiltration and related immune functions, combined with drug sensitivity analysis of common PCa treatments, suggested that the risk model could effectively guide clinical therapy for PCa patients. Notably, the drug sensitivity analysis included AZD5363 and docetaxel, both of which are included in the ProCAID clinical trial, which indicates potential benefits for guiding related clinical studies (Crabb et al., 2017). Although our drug sensitivity analysis did not identify the key drugs used in androgen deprivation therapy (ADT) for prostate cancer, AZD5363 combined with docetaxel has shown an increase in median overall survival (mOS) in metastatic castration-resistant prostate cancer (mCRPC). This potentially indicates that our model has a unique predictive value for the mCRPC population (Simon et al., 2022). Furthermore, we conducted an in-depth analysis of the protective gene MPO within the risk model, which highlights its association with immune cell subpopulations and confirms its crucial role as a lactate-related gene that can influence PCa cell migration and invasion.

Our GO analysis of the nine mitochondria-related genes revealed that the genes were associated with muscle development, adaptation, and function, particularly at the levels of skeletal and striated muscle. These functions are crucial for organismal movement and environmental adaptation (Stefano et al., 2011), which may be linked to the metabolic plasticity of tumors. The CC terms were enriched in specific muscle cell structures, and the MF terms were related primarily to muscle contraction and the regulation of muscle structure. This indicates that the key genes in the risk model are involved in motor function and metabolic regulation (Olivença, 2023; Brendan et al., 2013). Mitochondria are the primary source of ATP during muscle contraction and maintenance. During intense exercise and in cases of mitochondrial dysfunction, lactate accumulates through anaerobic glycolysis, which provides additional energy to the muscle. However, lactate accumulation can lead to an acidic environment, which affects calcium channels and muscle protein function; this in turn alters the microenvironment and potentially induces tumor development (Ann et al., 2006; George and Brooks, 2018; Wu Danchen et al., 2021). Our findings align with the current understanding of metabolic reprogramming in cancer and extend this understanding, particularly in the context of PCa. For example, Wang et al. reported that lactate acts as a signaling molecule to promote tumor immune evasion by modulating immune cell function (Anushka et al., 2019). Our study further enriches this field by identifying the relevance of specific lactate metabolism- and mitochondria-related genes in the prognosis of PCa.

The identification and validation of LMRGs as prognostic biomarkers for PCa is the most significant contribution of this study. By constructing robust LMRGs, we not only provide a novel tool for stratifying patients according to risk, but we also identify potential targets for therapeutic intervention. This study bridges the gap between metabolic reprogramming and clinical outcomes, offering a comprehensive understanding of how alterations in lactate metabolism and mitochondrial function drive PCa progression and impact patient survival. The validation of MPO as a key gene and its potential role as a protective factor in PCa further highlights the importance of these key targets and their metabolic pathways in cancer biology. In summary, our research contributes to the development of more personalized and effective treatment strategies for PCa.

Research has demonstrated that MPO is significantly linked to the risk of prostate cancer (PCa) through single nucleotide polymorphisms (Ding et al., 2013). MPO is a peroxidase that contains heme and catalyzes the formation of oxidants, such as hypochlorous acid (HOCl) and hypothiocyanous acid (HOSCN), from H_2_O^2^ and halide or pseudo-halide ions. These oxidants selectively oxidize proteins containing thiol groups, particularly those involved in the glycolytic pathway (Love et al., 2016; Lin et al., 2024). In this study, we revealed that MPO is connected to lactate metabolism and is vital in modulating lactate production by influencing the glycolytic pathway. This regulation leads to a marked reduction in migration, invasion, and EMT in PCa cells. The transforming growth factor-β (TGF-β) pathway plays a key role in fostering tumor metastasis and EMT across various tissue types (Anushka et al., 2019). Previous studies revealed that lactate can influence TGF-β-related pathways, thereby enhancing a tumor’s invasive characteristics (Baumann et al., 2009). For example, PCa cells can exploit lactate to promote PKM2/HIF-1-mediated transcriptional regulation and facilitate EMT (Giannoni et al., 2015). Therefore, substantial evidence indicates that lactate could be an upstream regulator of TGF-β, a crucial factor in EMT. The study by (Çakıcı et al., 2024) confirmed that reducing HIF-1α expression through the combination of inhibitors and chemotherapy drugs, thereby inhibiting the metabolic reprogramming of the EMT mechanism, can affect PC cell apoptosis and metastasis. Lactate enhances HIF-1α lactylation through the lactate transporter MCT-1, stimulating angiogenesis in PCa and influencing PCa proliferation and migration (Yongwen et al., 2022). Further research is needed to determine whether these mechanisms can form a conceptual and functional feedback loop in the tumorigenesis of PCa.

Furthermore, PCa cells that exhibit elevated MPO levels are sensitive to mitochondrial inhibitors. We treated PCa cells with antimycin A to disrupt electron transport, which resulted in mitochondrial dysfunction. Compared with control cells, which are vulnerable to the mitochondrial inhibitor antimycin A, LNCaP cells in which MPO was knocked down exhibited decreased sensitivity, migration, and invasion in response to this inhibitor. These data indicate that both mitochondrial metabolism and glycolysis may be required for PCa progression and metastasis. Compared with normal cells, cancer cells exhibit increased glycolysis due to the Warburg effect (Liberti et al., 2016). However, recent studies have shown that oxidative phosphorylation (OXPHOS) is as important as glycolysis in certain cancers (Wang and Patti, 2023). Several drugs, including metformin, atovaquone, and arsenic trioxide, are used clinically as OXPHOS inhibitors (Weinberg et al., 2015; Ashton et al., 2016; Wang et al., 2016). Therefore, in this study, we highlight novel applications of OXPHOS inhibitors in PCa cells with high MPO expression, highlighting their potential to improve therapeutic strategies for PCa management.

## Conclusion

In summary, this study is the first to construct a prognostic model for PCa based on LMRGs and provides effective guidance for the prognosis and drug treatment of PCa patients. Additionally, we investigated the role of the lactate-related gene MPO as a key factor that mediates lactate production by attenuating the glycolytic pathway, which leads to significant inhibition of migration, invasion, and EMT and increased drug sensitivity in PCa cells. The prognostic model and the MPO gene identified in this study not only offer insights into the metabolic basis of PCa but also present potential strategies for its treatment.

## Data Availability

The original contributions presented in the study are included in the article/Supplementary Material, further inquiries can be directed to the corresponding authors.
