# Supplementary figures and images for: Exploring the prognostic significance of lactate-mitochondria-related genes in prostate cancer
Source: Front Genet. 2025 Jan 6;15:1515045. doi: 10.3389/fgene.2024.1515045 (PMC11743670; doi:10.3389/fgene.2024.1515045)

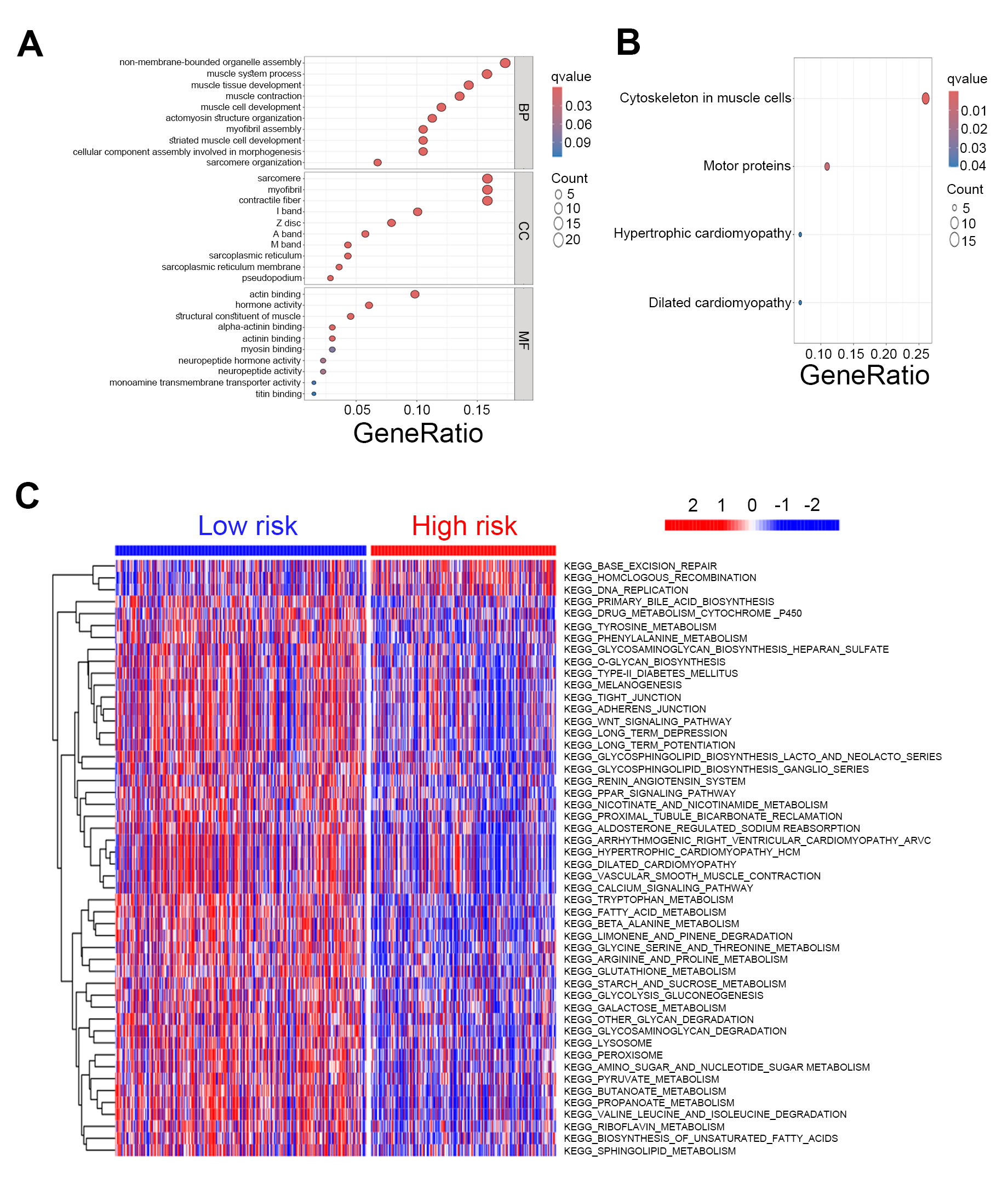

Supplement: Supplementary file 2 [file Image3.jpeg]

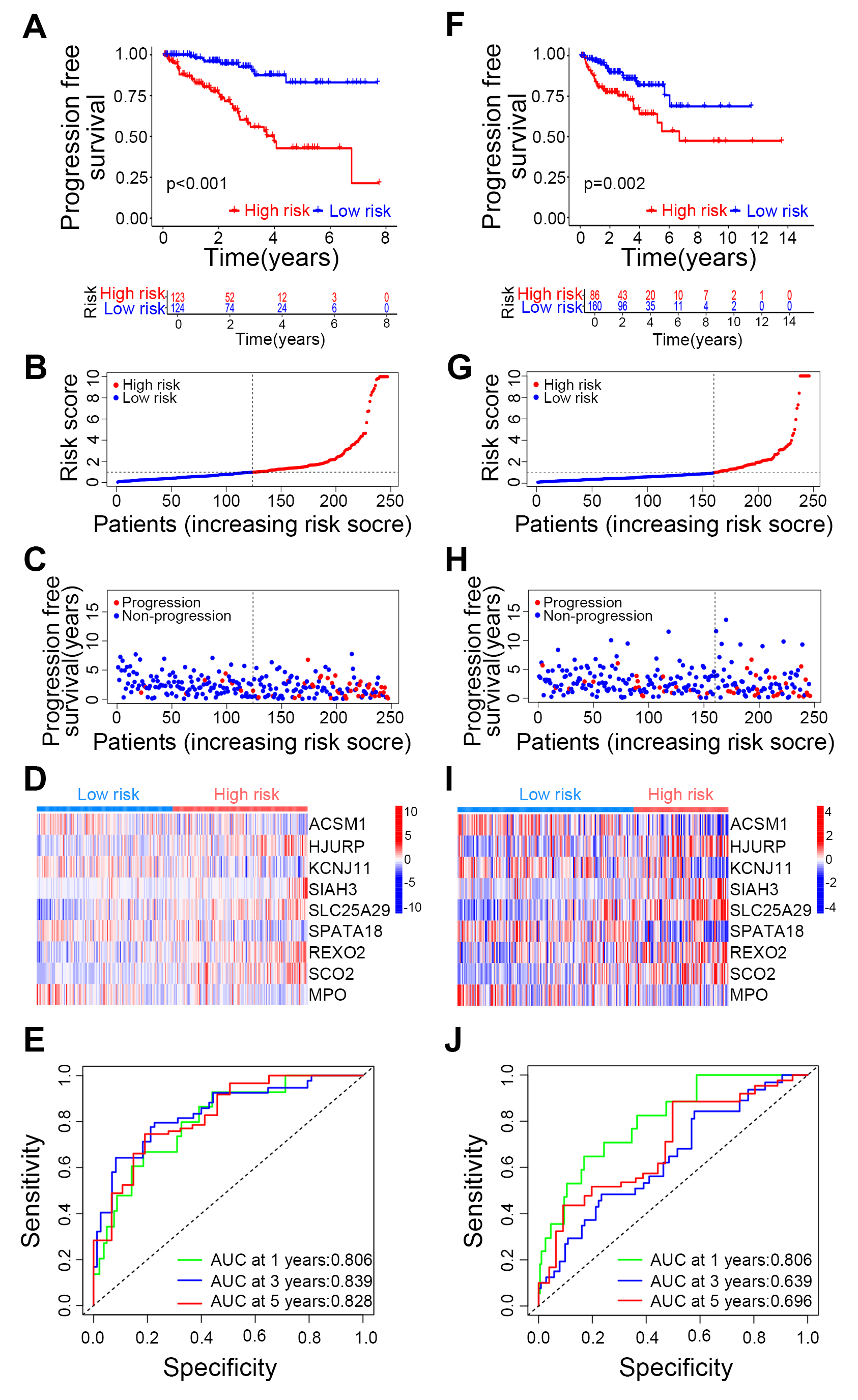

Supplement: Supplementary file 4 [file Image1.jpeg]

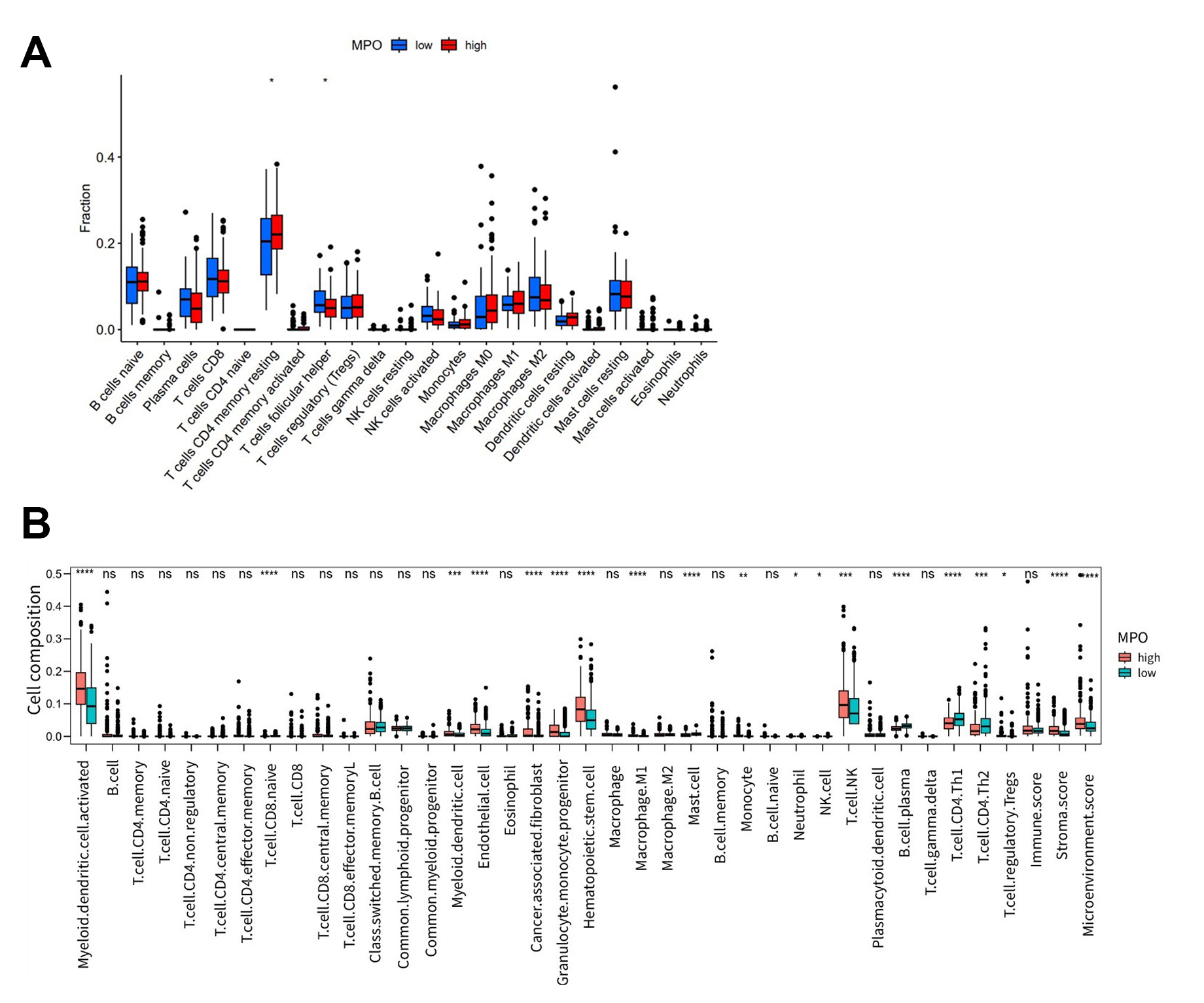

Supplement: Supplementary file 5 [file Image4.jpeg]

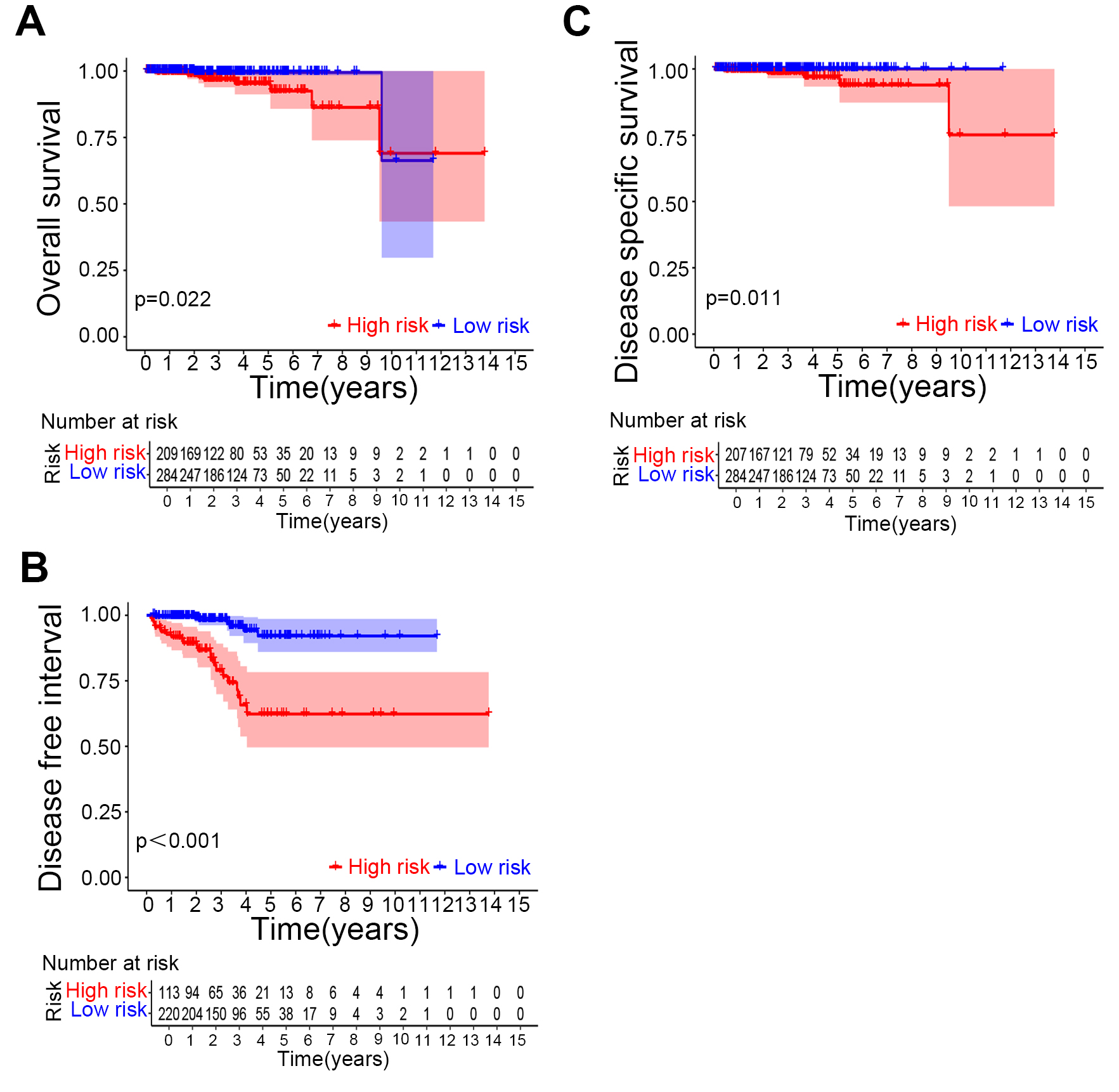

Supplement: Supplementary file 6 [file Image2.jpeg]

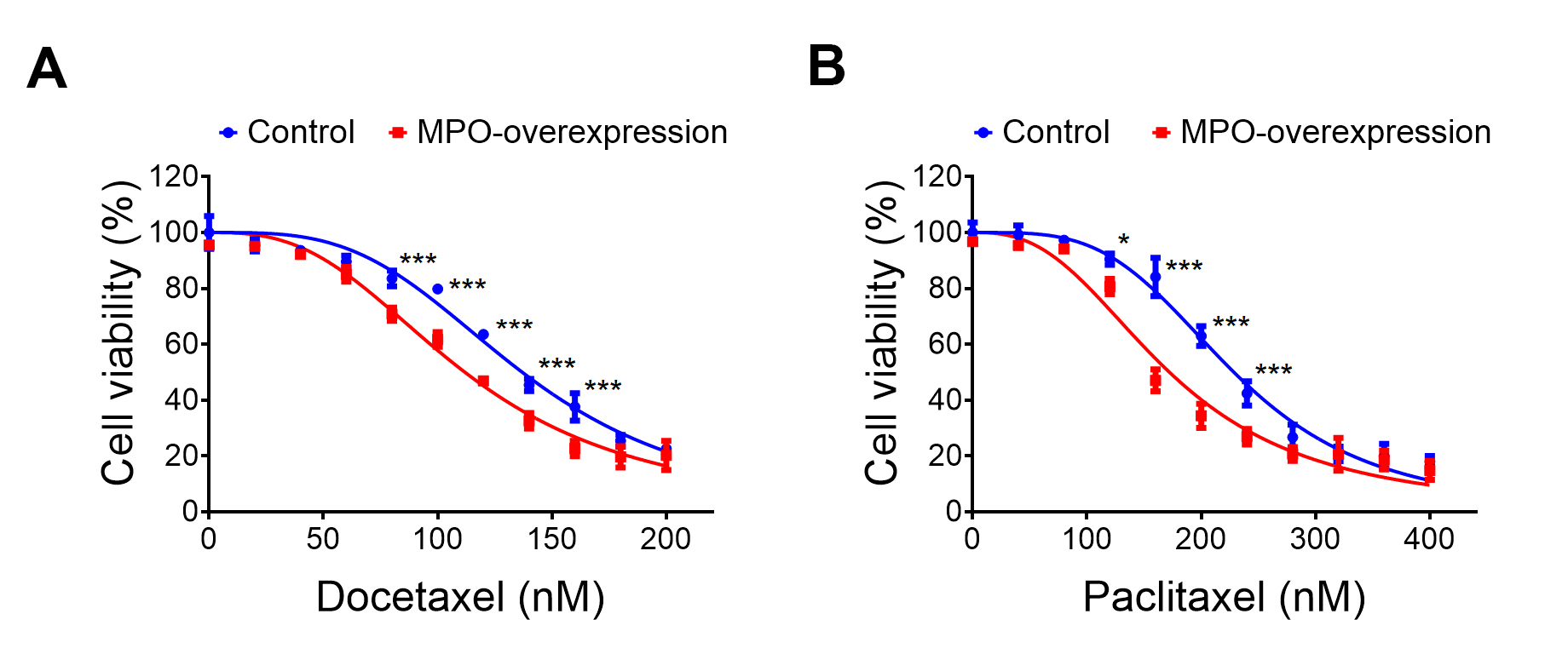

Supplement: Supplementary file 7 [file Image5.jpeg]
